# Autophagy Inhibition with Chloroquine Increased Pro-Apoptotic Potential of New Aziridine-Hydrazide Hydrazone Derivatives against Glioblastoma Cells

**DOI:** 10.3390/cells12141906

**Published:** 2023-07-21

**Authors:** Monika Witusik-Perkowska, Pola Głowacka, Adam M. Pieczonka, Ewa Świderska, Agnieszka Pudlarz, Michał Rachwalski, Julia Szymańska, Magdalena Zakrzewska, Dariusz J. Jaskólski, Janusz Szemraj

**Affiliations:** 1 Department of Medical Biochemistry, Medical University of Lodz, 6/8 Mazowiecka Str., 92-215 Lodz, Poland; pola.glowacka@stud.umed.lodz.pl (P.G.); ewa.swiderska@stud.umed.lodz.pl (E.Ś.); agnieszka.pudlarz@umed.lodz.pl (A.P.); janusz.szemraj@umed.lodz.pl (J.S.); 2 Department of Organic and Applied Chemistry, Faculty of Chemistry, University of Lodz, Tamka 12, 91-403 Lodz, Poland; adam.pieczonka@chemia.uni.lodz.pl (A.M.P.); michal.rachwalski@chemia.uni.lodz.pl (M.R.); julia.szymanska@edu.uni.lodz.pl (J.S.); 3 Department of Molecular Pathology and Neuropathology, Medical University of Lodz, Pomorska 251, 92-216 Lodz, Poland; magdalena.zakrzewska@umed.lodz.pl; 4 Department of Neurosurgery and Neurooncology, Medical University of Lodz, Barlicki University Hospital, Kopcinskiego 22, 90-153 Lodz, Poland; dariusz.jaskolski@umed.lodz.pl

**Keywords:** autophagy, apoptosis, tumor therapy escape, glioblastoma, aziridine derivatives, hydrazide hydrazone derivatives, chloroquine

## Abstract

Tumor therapy escape due to undesired side effects induced by treatment, such as prosurvival autophagy or cellular senescence, is one of the key mechanisms of resistance that eventually leads to tumor dormancy and recurrence. Glioblastoma is the most frequent and practically incurable neoplasm of the central nervous system; thus, new treatment modalities have been investigated to find a solution more effective than the currently applied standards based on temozolomide. The present study examined the newly synthesized compounds of aziridine–hydrazide hydrazone derivatives to determine their antineoplastic potential against glioblastoma cells in vitro. Although the output of our investigation clearly demonstrates their proapoptotic activity, the cytotoxic effect appeared to be blocked by treatment-induced autophagy, the phenomenon also detected in the case of temozolomide action. The addition of an autophagy inhibitor, chloroquine, resulted in a significant increase in apoptosis triggered by the tested compounds, as well as temozolomide. The new aziridine–hydrazide hydrazone derivatives, which present cytotoxic potential against glioblastoma cells comparable to or even higher than that of temozolomide, show promising results and, thus, should be further investigated as antineoplastic agents. Moreover, our findings suggest that the combination of an apoptosis inducer with an autophagy inhibitor could optimize chemotherapeutic efficiency, and the addition of an autophagy inhibitor should be considered as an optional adjunctive therapy minimizing the risk of tumor escape from treatment.

## 1. Introduction

Glioblastoma (GB) is the most common aggressive and lethal tumor of the central nervous system (CNS), accounting for 15% of all CNS tumors and about 45% of primary malignant brain tumors. Despite treatment, only a small fraction of patients (up to 20%) survive two years from the initial diagnosis, while the five-year survival rate for GB is limited to 5%. Current optimal therapy results in a modest 14-month overall median survival in patients undergoing maximum safe resection and adjuvant chemo- and radiotherapy. In spite of great efforts taken by scientists in the field of translational research, the prognosis for GB remains extremely poor since realistic curative treatment is still unavailable [1].

The reasons underlying drug-resistance of GB are diverse and include, among others, diffuse and infiltrative growth, the intra-heterogeneous nature of tumor that consists of molecularly different cells, rapid proliferative rate, presence of tumor stem cells, dysfunctional DNA damage repair mechanisms, the dynamically switching phenotype of neoplastic cells [2,3,4,5]. Moreover, the initial therapy often induces processes hindering the desired response to therapy, such as pro-survival autophagy and apoptosis inhibition, and cell senescence, leading to tumor dormancy and subsequent remodeling to a more malignant phenotype and senescence-associated secretory phenotype (SASP) promoting a variety of aspects of tumorigenesis [6,7,8]. Consequently, those mechanisms may result in tumor therapy escape and expansion of resistant cell populations, which eventually leads to GB recurrence.

Considering the current advances in the field of GB treatment, further efforts aimed at searching for new therapeutic options are fully justified. Although a variety of potential drugs have been investigated so far, temozolomide (TMZ) is still the first-choice chemotherapeutic in GB. However, the development of resistance to this agent often limits the effective treatment [9].

Apart from the most intensively investigated candidate drugs, hydrazide hydrazone-derivatives and aziridine-derivatives are two classes of chemicals presenting anti-tumor potential, including anti-proliferative and apoptogenic properties [10,11,12,13,14]. Their anti-neoplastic action was revealed against several neoplastic cell lines, including lung carcinoma, pancreatic carcinoma, breast cancer, liver cancer, ovarian cancer, prostate cancer, renal cancer, and hematological tumors in vitro. The recognized mechanisms underlying the anti-tumor action of these agents comprise oxidative stress generation, EGFR inhibitory activity, cell cycle arrest, inhibition of tubulin polymerization, and genotoxic potential (e.g., via alkylating properties) [13,15,16,17,18]. However, a review of a modest amount of published data suggests that the molecular background of their anti-cancer activity depends on the chemical structure of particular agents and the type of neoplastic cells.

Aziridines are widely used as building blocks in multi-step syntheses of more complex molecules; however, due to the presence of the aziridine ring, their derivatives exhibit significant biological activity [19]. To obtain the anti-cancer activity of new potential drugs containing the aziridine ring, we additionally introduced the N-acylhydrazone group (NAH), another pharmacophore fragment known in the literature [20]. This moiety is crucial in the synthesis of drugs having anti-cancer properties, and it also produces clinically proven effects. Currently, there are preparations based on the NAH derivatives available on the market, such as nitrofurazone, nitrofuraxide, or dantrolene [21].

To the best of our knowledge, the classes of compounds based on hydrazide hydrazone (NAH) and aziridine derivatives have not yet been tested on a neoplastic model of CNS tumors. Therefore, our study is aimed at investigating the anti-tumor potential of newly synthesized compounds of aziridine–hydrazide hydrazone derivatives with the use of GB-derived in vitro models.

## 2. Materials and Methods

### 2.1. In Vitro Models of Glioblastoma and Non-Neoplastic Cells (NHA)

Tumor samples were obtained from the Department of Neurosurgery and Oncology of the Central Nervous System, Medical University of Lodz, Poland. All the procedures were performed in accordance with the ethical standards of the Bioethics Committee of the Medical University of Lodz (Approval Ref. No. RNN/148/08/KE and RNN/160/15/KE) and informed consent was obtained from all the patients, in compliance with the Declaration of Helsinki. According to the WHO criteria, the tumor samples intended for cell culture generation were classified as glioblastoma, NOS/not otherwise specified (the mutational status of the *IDH* gene family was not verified) [22]. Glioblastoma cultures were derived from three tumor samples obtained from three patients according to the procedure of culture generation described in our previous reports [23,24]. Subsequently, the GB cells were cultured as an adherent model in serum-supplemented medium (DMEM/F12, Gibco, Life Technologies Europe B.V., Bleiswijk, The Netherlands; with 10% FBS, Gibco, Life Technologies Limited, Paisley, United Kingdom; and antibiotics: Gentamycin solution and Penicilin-Streptomycin, Sigma-Aldrich, St. Louis, MO, USA). Depending on proliferation activity, cells were passaged to a new culture dish every 3–5 days and expanded for further analysis. The neoplastic status of cells was verified according to rules previously described and based on expression of astrocytoma-associated antigens IL13Rα2, Fra-1, and EphA2 [23].

Commercially available normal human astrocytes (NHA) were used as non-neoplastic control cells. NHA were cultured according to manufactures protocol (Lonza, Basel, Switzerland).

### 2.2. Synthesis of Tested Compounds

General: Melting points were determined in a capillary using a STUART SMP30 and were uncorrected. The ^1^H (600 MHz) and ^13^C{1H} (150 MHz) spectra were measured on a Bruker Avance III instrument (Bruker, Billerica, MA, USA) using solvent signals as reference. Chemical shifts (δ) were given in ppm and coupling constants J in Hz. Assignments of signals in ^13^C-NMR spectra were made based on HMQC experiments. HR-MS: Bruker Esquire LC spectrometers (Bruker Daltonics, Billerica, MA, USA). All the solvents are commercially available reagents and were used as received. Aziridine carbohydrazide was obtained according to a published procedure [25].

The general procedure for the synthesis of chiral hydrazones ARA12 and ARA13 from aziridine-2-carbohydrazide was the following: an equimolar quantity of appropriate aldehyde was added to a stirred solution of an aziridine-2-carbohydrazide (1 mmol) in ethanol (5 mL) at 20 °C; the mixture was refluxed for three hours, the solution was concentrated, and the products were purified by the crystallization from methanol.

(2S)-N-[(E)-[4-(Trifluoromethyl)phenyl]methyleneamino]-1-trityl-aziridine-2-carboxamide (ARA12): colorless oil; yield: 77%. ^1^H-NMR (600 MHz, CDCl_3_, δ, ppm): 9.76 (1H, br. s, NH); 8.36 (1H, s, =CH); 7.82–7.58 (4H, 2m, 4 arom. H); 7.40–7.19 (15H, 3m, 15 arom. H); 2.15 (1H, dd, J = 2.7 Hz, J = 6.6 Hz, 1 aziridine H); 2.08 (1H, d, J = 2.7 Hz, 1 aziridine H); 1.55 (1H, d, J = 6.6 Hz, 1 aziridine H). ^13^C-NMR (150 MHz, CDCl3, δ, ppm): 167.0 (C=O); 146.9 (C=N); 142.9, 129.5, 129.3, 128.0, 127.9, 127.6, 127.5, 126.9, 125.7 (Carom); 74.9 (C-N); 33.9, 30.2 (Caziridine). HR-EI-MS: 498.1871 (M+, C_30_H_23_F_3_N_3_O^+^; calcd. 498.1871).

(2S)-N-[(Z)-(5-fluoro-2-hydroxyphenyl) methyleneamino]-1-trityl-aziridine-2-carboxamide (ARA13): colorless crystals; yield: 50%; m.p. 115–117 °C (MeOH). ^1^H-NMR (600 MHz, CDCl_3_, δ, ppm): 10.66 (1H, br.s, OH); 9.60 (1H, br.s, NH); 8.46 (1H, s, N=CH); 7.40–7.20 (15H, 4m, 15 arom. H); 6.99–6.86 (3H, 2m, 3 arom H); 2.12 (1H, ddd, J = 2.7 Hz, J = 6.6 Hz, 1H); 2.08 (1H, d, J = 2.7 Hz, 1H); 1.56 (1H, d, J = 6.6 Hz, 1H). ^13^C-NMR (150 MHz, CDCl_3_, δ, ppm): 166.5 (C=O); 144.5 (C=N); 142.8, 129.6, 129.3, 128.7, 128.1, 127.9, 127.8, 127.7, 127.5, 126.8 (Carom); 74.7 (C-N); 33.8, 30.2 (Caziridine). HR-EI-MS: 464.1851 (M+, C_29_H_24_FN_3_O_2_^+^; calcd. 464.1852).

### 2.3. Drug Cytotoxicity Assay

The Cell Counting Kit-8 (CCK-8) assay (Sigma-Aldrich, St. louis, MO, USA) was used to measure the cytotoxicity of the tested drugs on glioblastoma cells. The amount of the formazan dye generated by the activity of cellular dehydrogenases is directly proportional to the number of living cells.

Glioblastoma cells and NHA cells cultured under traditional adherent conditions (5 × 10^3^ cells/mL) were grown in 96-well plates for 24 h and treated with the tested compounds (six new synthesized compounds of aziridine–hydrazone derivatives) over a range of concentrations (0, 25, 50, 100, 150, 200, 300, 500 μg/mL).

After 48 h, the extent of cell growth was assessed using the CCK-8 assay. The CCK-8 solution (10 μL) was added to each well, followed by incubation for 3 h at 37 °C. The absorbance of the cell culture medium at 450 nm was determined by a multiplate reader (Glomax Multi Detection System; Promega, Madison, WI, USA). Cell viability was expressed as a percentage of that of the control (untreated) cells. All the results obtained in the study were based on at least three experiments.

### 2.4. Assay for Cell Apoptosis by Annexin V/PI Double Staining

The extent of apoptosis/necrosis was measured using the FITC Annexin V Apoptosis Detection Kit I, (BD Biosciences, Erembodegem, Belgium) according to the manufacturer’s instructions. In brief, the glioblastoma cells were plated at a seeding density of 1 × 10^5^ cells/mL and treated with different concentrations of the tested drugs (ARA12: 0, 100, 150, 200 μg/mL; ARA13: 0, 100, 200, 300 μg/mL; TMZ: 500 μM) for 48 h and 96 h. After the incubation period, cells were harvested and washed twice with PBS and stained with annexin V-FITC/propidium iodide (PI) at room temperature in the dark for 15 min, according to the manufacturer’s instructions. Data acquisition and analysis were performed by flow cytometry (CytoFLEX; Beckman Coulter Inc., Brea, CA, USA). The four populations of cells were analyzed. Viable cells were negative for both PI and annexin V; early-stage apoptotic cells were positive for annexin V and negative for PI; late-stage apoptotic cells were positive for both annexin V and PI, and necrotic cells were positive for PI. The results are presented as the means of percentage of apoptotic cells from at least three independent experiments.

### 2.5. Assay for Autophagy Detection by FACS with the Use of Autophagy Detection Kit

According to the manufacturer (Abcam, Cambridge, MA, USA), the Autophagy Detection Kit (ab139484) measures autophagic vacuoles and monitors autophagic flux in living cells using a dye that selectively labels autophagic vacuoles. The cationic amphiphilic tracer dye (Green Detection Reagent) was optimized through the identification of titratable functional moieties that allow for minimal staining of lysosomes while exhibiting bright fluorescence upon incorporation into pre-autophagosomes, autophagosomes, and autolysosomes (autophagolysosomes). The assay offers a rapid and quantitative approach to monitoring autophagy in living cells. The glioblastoma cells were plated at a seeding density of 1 × 10^5^ cells/mL and treated with the tested drugs alone (ARA12—150 μg/mL, ARA13—200 μg/mL, TMZ—500 μM) or in combination with a known autophagy inhibitor, chloroquine (CQ—10 μM).

After 48 h, the cells were harvested and processed according to the manufacturer’s protocol (Abcam). The control and treated cells were incubated for 30 min at 37 °C with Green Detection Reagent (GDR). Data acquisition and analysis were performed by flow cytometry (CytoFLEX; Beckman Coulter Inc., Brea, CA, USA). Finally, the results yielded as mean fluorescence intensity (MFI) were expressed as autophagy activity factor (AAF) calculated according to the following equation:

AAF = 100 × (MFItreated − MFIcontrol/MFItreated).

Fluorescence microscopy images of control and drug-treated cells were performed using an Olympus BX-41 fluorescence microscope to validate the staining capability of Green Detection Reagent (autophagy dye). The analyses were performed as previously described [24,26].

### 2.6. Immunofluorescence Analysis

The glioblastoma cells were plated at a seeding density of 2.5 × 10^4^ cells/well and treated with tested drugs alone or in combination with CQ (ARA12—150 μg/mL, ARA13—200 μg/mL, TMZ: 500 μM; CQ—10 μM). After 48 h of treatment, the cells were fixed with 4% PFA for 15 min and subjected to immunofluorescence analysis. The cells were permeabilized with 0.1% Triton X-100 for 10 min. Non-specific binding sites were blocked with 2% donkey serum in PBS for 1 h. Subsequently, the cells were incubated for 2 h with the primary antibodies (1:500, anti-LC3B rabbit polyclonal antibody, SAB5700240, Sigma-Aldrich; 1:500, mouse monoclonal Anti-Caspase3 (active form) Antibody; MAB10753; Sigma-Aldrich). For visualization, the appropriate species-specific fluorochrome-conjugated secondary antibodies (1:500 donkey anti-rabbit Alexa-Fluor488; 1:500, donkey anti-mouse Alexa-Fluor594; Molecular Probes) were applied for 1 h in the dark. Controls were created with secondary antibodies alone and matched isotype controls in place of the primary antibodies, and these were processed in the same manner. Slides were mounted with ProLongGold Antifade Reagent with DAPI (Molecular Probes, Invitrogen, Eugene, Oregon, USA), coverslipped, and examined using an Olympus BX-41 fluorescence microscope. Microphotographs of DAPI staining were also used to show cells presenting apoptotic hallmarks.

Immunofluorescence results of caspase-3 active form detection were subjected to semiquantitative analysis based on assessment of mean fluorescence intensity of particular cells (for each sample, signal from at least 100 cells were counted from 3 separate microphotographs performed with the same exposure time). The data were presented as histograms showing distribution of cells presenting different levels of fluorescence intensity.

### 2.7. Western Blot Analysis

Glioblastoma cells of each patient-derived cell line were harvested from samples comprising about 5 × 10^5^ cells incubated for 48 h in the following conditions: untreated cells with no additions, incubated with 500 μM TMZ, incubated with 500 μM TMZ and 10 μM CQ, incubated with ARA12—150 μg/mL, incubated with 150 μg/mL ARA12 and 10 μM CQ, incubated with 200 μg/mL ARA13, incubated with 200 μg/mL ARA13 and 10 μM CQ. The protein extracts from cell cultures was obtained with the use of RIPA buffer (SERVA). Equal amounts of protein extracts (30 μg) were separated by gradient 8–16% SDS-PAGE on ExpressPlus™ PAGE Gels (GenScript, Piscataway, NJ, USA) before being transferred onto 0.2 µm PVDF membranes with Trans-Blot Turbo RTA Mini 0.2 µm PVDF Transfer Kit (Bio-Rad, Hercules, CA, USA) using the Trans-Blot^®^ Turbo™ Transfer System (Bio-Rad, Hercules, CA, USA). Nonspecific binding sites were blocked by incubating the membranes 1h at RT in TBST buffer (TRIS Buffered Saline with Tween 20; pH 7.6) containing 5% non-fat milk powder. The membranes were then incubated 2 h at RT with anti-LC3B rabbit polyclonal antibody (Sigma-Aldrich, 1:1000 dilution) for LC3B visualization and anti- β-actin rabbit polyclonal antibody (Invitrogen, Waltham, MA, USA; dilution 1:1000). Then, the membranes were extensively washed in TBST buffer and incubated for 1 h at RT with goat anti-mouse horseradish peroxydase-conjugated secondary antibody or goat anti-rabbit horseradish peroxydase-conjugated secondary antibody (Invitrogen, Waltham, MA, USA; dilution 1:10,000). After washing in TBST buffer, antigen–antibody complexes were detected by enhanced chemiluminescence, using Pierce™ ECL Western Blotting Substrate (Thermo Scientific, Rockfold, IL, USA) according to the manufacturer’s instructions.

Western blot results were subjected to densitometric analysis to assess autophagic flux based on LC3-II turnover according to previously described method [27,28,29].

### 2.8. Detection of subG0/G1 Phase

In order to confirm apoptosis occurrence, subG0/G1 phase was detected by flow cytometry analysis of cells processed with Cycle Analysis Kit (BioVision Inc., Milpitas, CA, USA) based on propidium iodide staining. GB cells were seeded at 1 × 10^5^/well density and, after 24 h of growing, were incubated with TMZ (500 µg/mL), ARA12 (150 µg/mL), or ARA13 (200 µg/mL) for 48 h. After the treatment, cells were harvested, prepared according to manufacturer’s instruction, and then subjected to flow cytometry analysis.

### 2.9. Statistical Analysis

All the experiments were repeated at least three times, and the data were expressed as the means ± SD. Comparisons between the two groups were performed using Student’s *t*-test, and differences among three or more groups were determined using one-way analysis of variance (ANOVA) followed by Holms–Sidak post hoc test. Multiplicity adjusted value of *p* < 0.05 was considered statistically significant.

## 3. Results

### 3.1. Synthesis of New Aziridine–Hydrazide Hydrazone Derivatives and Initial Screening of Their Anti-Tumor Activity

A typical procedure for the synthesis of carboxylic acid hydrazide derivatives is the treatment of the corresponding carbohydrazide with diverse electrophilic agents. As previously described, aziridine-2-carbohydrazide can be easily obtained from methyl (*S*)-N-triphenylmethyl aziridinate in the reaction with hydrazine hydrate [25]. The reaction of aziridine carbohydrazide with aromatic aldehydes in boiling methanol afforded the expected enantiopure hydrazide hydrazones ARA12 and ARA13. Their structures were confirmed based on spectroscopic data; optical purity was established by HPLC using the Chiralpak AD-H column.

**Figure 1:**
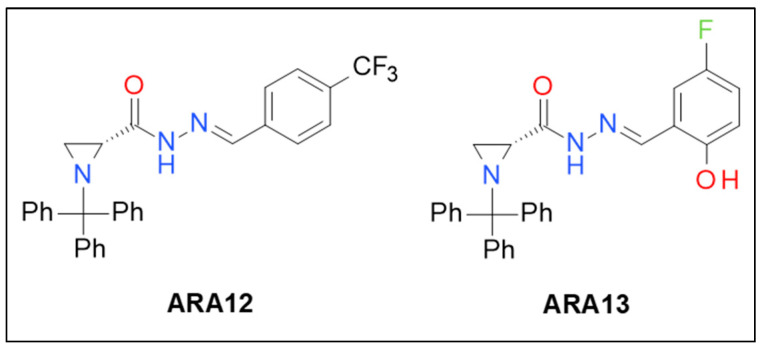
Structures of aziridine–hydrazide hydrazones with anti-tumor activity (ARA12; ARA13).

Initial screening of newly synthesized aziridine–hydrazide hydrazone derivatives on GB cells in vitro allowed to single out two compounds presenting cytotoxic potential against tumor cells—ARA12 and ARA13. Figure 1 presents structures of the most promising derivatives selected for further research on their anti-neoplastic potential.

To evaluate the utility of these agents as candidate drugs for the therapy of CNS tumors, the BBB Scores were calculated for both examined aziridine–hydrazide hydrazone derivatives. The BBB Score is an algorithm to assess the probability of an investigated drug passing the blood–brain barrier based on the drug’s physicochemical properties. The obtained BBB Score values were 3.66 for ARA12 and 3.40 for ARA13 [30].

### 3.2. Newly Synthesized Aziridine–Hydrazide Hydrazone Derivatives Present Proapoptotic Potential against GB Cells Higher than TMZ

To evaluate the anti-neoplastic activity of the newly synthesized aziridine–hydrazide hydrazone derivatives, the three GB cell lines (G16, G114, G116) established previously from resected tumors were employed.

**Figure 2:**
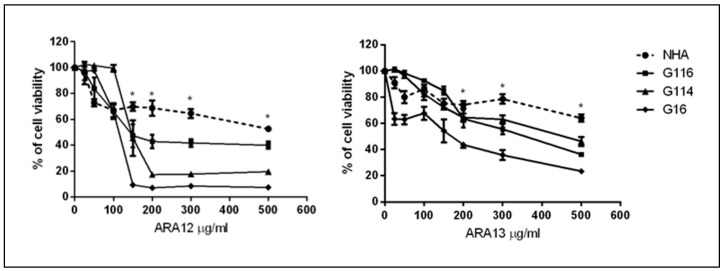
Dose-dependent response of GB cells and NHA treated with new aziridine–hydrazide hydrazone derivatives—ARA12 and ARA13. Results of CCK-8 assay revealed cytotoxic potential of ARA12 and ARA13 against GB cells after a 48-h treatment; however, individual tumors (G114, G116, G16) presented differences in treatment sensitivity. Starting from concentration of 150 µg/mL for ARA12 and 200 µg/mL for ARA13, all the tested GB cell lines have a significantly higher sensitivity to the applied agents than normal human astrocytes (NHA); *p* < 0.05 (*).

Since the results of the viability assay (CCK 8 test) performed for the three GB-derived cell lines (G16, G114, G116) revealed differences in the treatment response between particular tumors, the concentrations of 100, 150, 200 µg/mL for ARA12 and 100, 200, 300 µg/mL for ARA13 were selected for further experiments for all the tested GB cell lines. As compared to normal human astrocytes (NHA), both compounds presented a stronger cytotoxic effect on tumoral cells; however, a greater difference was detected for ARA12. Starting from a concentration point of 150 µg/mL for ARA12 and 200 µg/mL for ARA13, all the tested GB cell lines have a significantly higher sensitivity to the applied treatment than NHA; *p* < 0.05 (Figure 2).

To evaluate the proapoptotic potential of selected aziridine–hydrazide hydrazone derivatives, FACS analyses based on annexin V/PI staining were performed. Cytotoxicity of examined compounds was initially assessed for GB cells incubated with ARA12 (150 µg/mL) and ARA13 (200 µg/mL) for 24 h. Results obtained for samples derived from two of the three tumors have shown no difference in cell viability when compared to untreated cells (results available in Supplementary Material—Table S1). Therefore 48-h and 96-h incubation periods were applied in further investigations. The GB cells were subjected to treatment for 48 h with the three concentrations of ARA12 (100, 150, 200 µg/mL), ARA13 (100, 200, 300 µg/mL), and TMZ (500 µM) as the standard drug used in GB therapy.

**Figure 3:**
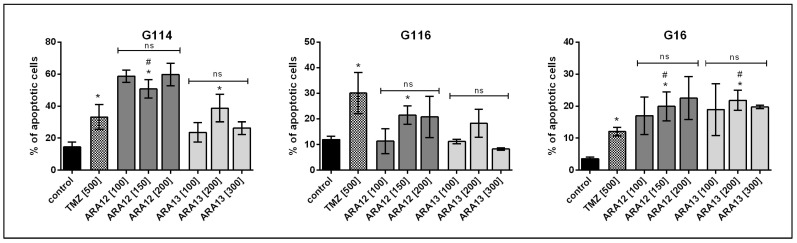
Concentration-dependent analysis of viability of GB cells after a 48-h treatment with aziridine-hydrazide hydrazone derivatives (ARA12, ARA13) and TMZ—results of apoptosis assay (annexin V/PI staining) based on FC analysis for G114, G116, and G16 tumors. ARA12 (150 µg/mL) and ARA13 (200 µg/mL) induced apoptosis of GB cells within 48 h, yielding a significantly higher number of apoptotic cells as compared to untreated control, except from G116 treated with ARA13 (*); *p* < 0.05. Dose increase of ARA12 (100, 150, 200 µg/mL) and ARA13 (100, 200, 300 µg/mL) did not result in any significant changes in apoptotic cell percentage (ns). Aziridine–hydrazide hydrazone derivatives (ARA12—150 µg/mL and ARA13—200 µg/mL) demonstrate a similar or even stronger pro-apoptotic activity than that of TMZ (500 µM) in the case of G16 and G114 tumors (#); *p* < 0.05.

For both tested compounds, ARA12 and ARA13, the dose-dependent analysis did not reveal any significant changes in apoptosis efficiency after 48-h treatment (*p* < 0.05). The percentage of apoptotic cells did not exceed the threshold of 25% in the case of G16 and G116. G114 tumor cells presented higher sensitivity to the applied treatment, which resulted in about 50% of apoptotic cells for ARA12 and close to 40% of apoptotic cells for ARA13. After 48 h, the proapoptotic potential of ARA12 and ARA13 was similar to or even higher (*p* < 0.05) than the activity of TMZ as a drug routinely used in GB treatment; however, a slight difference between the tumors was observed (Figure 3).

Since the dose increase did not result in any significant cytotoxicity enhancement, the time of treatment was prolonged to 96 h. GB cells were subjected to treatment with ARA12 at a concentration of 150 µg/mL, ARA13 at a concentration of 200 µg/mL, and TMZ at a dose of 500 µM.

**Figure 4:**
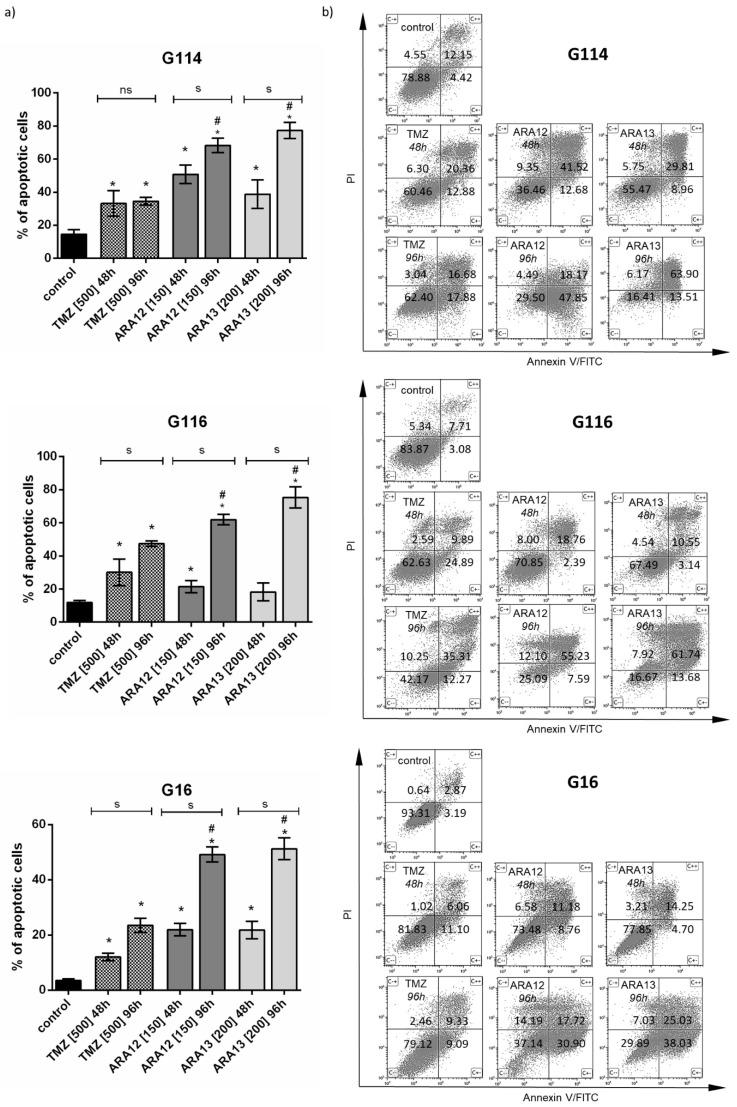
Time-dependent analysis of viability of GB cells after 48- and 96-h treatments with aziridine–hydrazide hydrazone derivatives (ARA12, ARA13) and TMZ—results of apoptosis assay (annexin V/PI staining) based on FACS analysis for G114, G116, and G16 tumors. (**a**) Analysis of apoptosis efficiency after a 96 h treatment with ARA12 and ARA13 revealed significantly higher percentage of apoptotic cells for each tumor as compared to untreated control (*) and to the status detected after a 48-h treatment (s); *p* < 0.05; ns—*p* > 0.05. Pro-apoptotic potential of aziridine–hydrazide hydrazone derivatives expressed as percentage of apoptotic cells was also compared to TMZ, yielding significant results for all tumors after 96-h treatment (#); *p* < 0.05. (**b**) Results of FACS analysis show response of G114, G116, and G16 tumors to the tested agents (TMZ, ARA12, ARA13) after 48- and 96-h treatments (mean percentage values of cells for distinguished populations were provided).

The time-dependent analysis resulted in a marked increase in treatment efficiency both in the case of ARA12 and ARA13. Overall, the percentage of apoptotic cells was about two to three times higher when compared to the period of the 48-h treatment (*p* < 0.05), finally reaching values of 50–70% for ARA12 and 50–80% for ARA13. An unexpected phenomenon was noted in the case of G114 treated with ARA12 when the number of late apoptotic cells after 48 h was higher than that detected after 96 h. This occurrence could be caused by the overgrowth of a more resistant cell population subsequently visible as early apoptotic fraction after 96 h of treatment; however, further investigation is needed to elucidate this phenomenon. Finally, after 96 h, both drug candidates presented significantly higher proapoptotic potential than TMZ (*p* < 0.05); (Figure 4).

**Figure 5:**
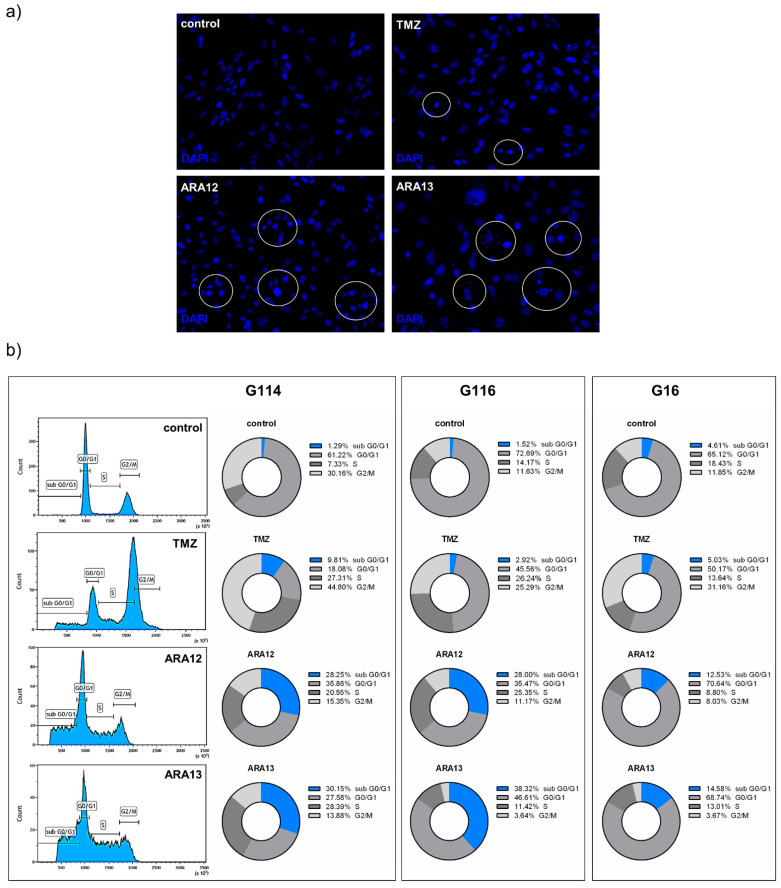
GB cells treated with new aziridine–hydrazide hydrazone derivatives (ARA12, ARA13) show hallmarks of apoptosis. (**a**) results of DAPI staining demonstrated presence of apoptotic cells (in circles) showing changes in the nuclear morphology—chromatin condensation and nuclear fragmentation in samples treated with ARA12 and ARA13, with weaker effects observed after TMZ treatment. (**b**) estimation of fractional DNA content by PI incorporation analyzed by FACS revealed presence of subG0/G1 population being the result of ARA12 and ARA13 treatment, while main effect of TMZ action was a block of cell cycle in G2/M phase after 48-h treatment.

The proapoptotic potential of the newly tested agents was also confirmed by the detection of cells presenting changes in nuclear morphology visualized with DAPI staining, as well as the occurrence of DNA fragmentation in cells belonging to subG0/G1 population identified via FACS analysis of cell cycle with propidium iodide (Figure 5). The method based on PI incorporation shows that ARA12 treatment generated a subG0/G1 fraction comprising about 28% of cells in the case of G114 and G116 and about 12% of cells in the case of G16 tumor. ARA13 treatment yielded about 30–38% of cells classified as subG0/G1 population in the case of G114 and G116 and close to 15% of cells presenting DNA fragmentation for G16 tumor. Analysis of TMZ-treated cells confirmed the ability of this drug to block the GB cell cycle in the G2/M phase, while the subG0/G1 fraction did not exceed 10% after 48 h for all analyzed tumors (Figure 5).

### 3.3. Inhibition of Treatment-Induced Autophagy Facilitate Apoptotic Cell Death of GB Cells

**Figure 6:**
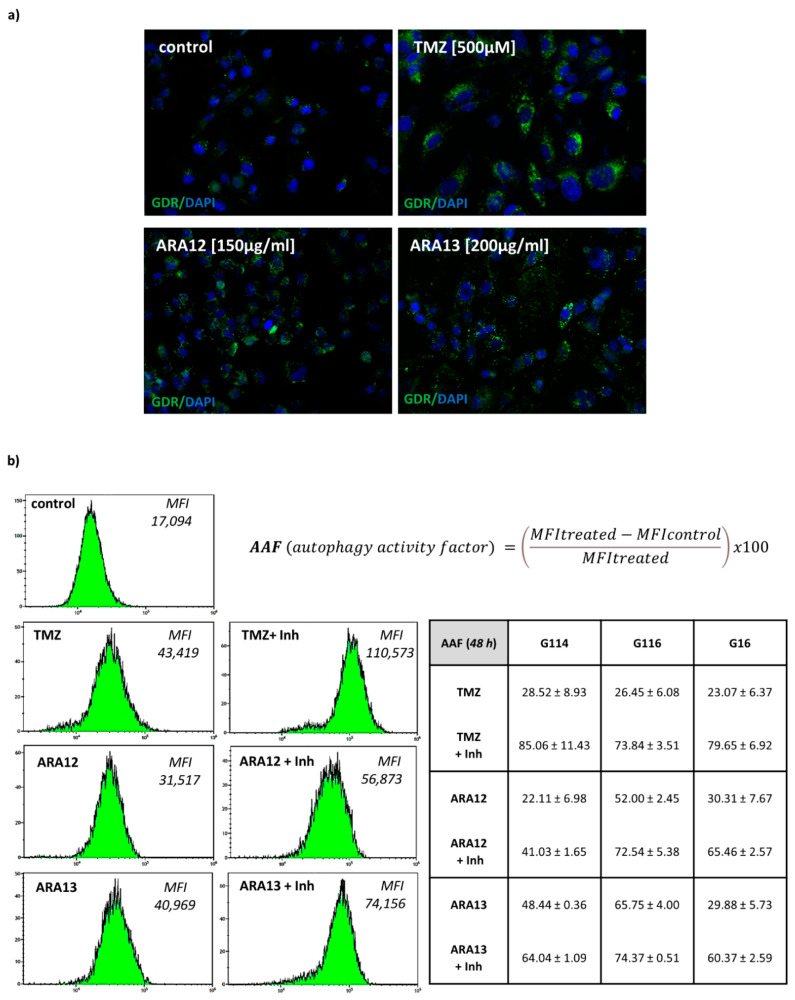
Autophagic response of GB cells treated with TMZ, ARA12, ARA13 and combination of the tested agents with an inhibitor: chloroquine; (**a**) Fluorescence microscopy images of GB cells stained with autophagy dye (GDR) after a 48-h treatment with the tested agents revealed ability of ARA12 and ARA13 to stimulate the autophagy process similarly to TMZ; (**b**) Output of FACS analysis demonstrated that CQ (Inh) addition results in blockage of the autophagy process in cells treated with TMZ, ARA12 or ARA13; the effect is visible as an increase in mean fluorescence intensity (MFI) of cells stained with GDR-sample results of autophagy assay for one tumor; quantitative analysis of FACS data for G114, G116, and G16 tumors, confirmed ability of ARA12 and ARA13 to induce autophagy in GB cells and demonstrated inhibition of this process in CQ presence.

In our previous study, we demonstrated that TMZ treatment induces autophagy in GB cells [24]. This phenomenon was confirmed in the present experiment. An autophagy assay also revealed that the newly tested compounds, ARA12 and ARA13, were able to stimulate autophagic flux in GB cells within 48 h at a level comparable to or even stronger than that of TMZ (Figure 6).

Data are expressed as autophagy activity factor: AAF = 100 × (MFItreated − MFIcontrol/MFItreated).

Since treatment-induced autophagy can hinder apoptosis, we hypothesized that autophagy inhibition may facilitate the activation of apoptotic cell death by the tested drug candidates. The GB cells were treated simultaneously with the tested compounds (ARA12, ARA13, and TMZ) and the well-known autophagy inhibitor, chloroquine (10 µM). Chloroquine blocks autophagic flux by impairing lysosomal function and autophagosome–lysosome fusion [31,32], causing accumulation of autophagosomes; thus, it could be detected as a higher autophagy activity factor (AAF). Quantitative analysis of autophagy assay by FACS confirmed this phenomenon, yielding higher values of AAF for GB cells subjected to treatment with a combination of the tested compounds (ARA12 or ARA13) and chloroquine. Similar results were obtained for GB cells treated with TMZ with the addition of the autophagy inhibitor (CQ) (Figure 6).

**Figure 7:**
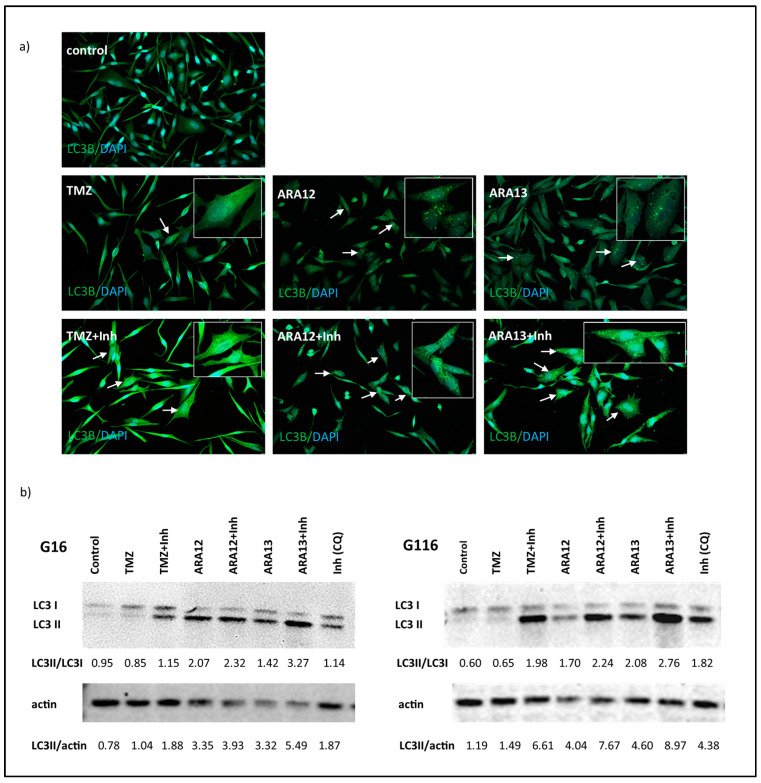
LC3II turnover confirms stimulation of autophagic flux by aziridine–hydrazide hydrazone derivatives (ARA12, ARA13). (**a**) Immunofluorescence analysis with anti-LC3 antibody shows presence of LC3 puncta (indicated by arrows and enlarged in boxes) related to autophagosome formation in GB cells treated with ARA12 and ARA13, combined treatment with CQ resulted in block of autophagy progress and accumulation of cells strongly positive for LC3 puncta. (**b**) Results of Western blot assay for LC3 protein followed by semiquantitative analysis confirmed autophagic flux stimulated by ARA12 and ARA13 (data presented for G16 and G116 tumors subjected to various modes of treatment; pictures of uncropped membranes were provided as Supplementary Figure S1a).

Additionally, autophagy occurrence in GB cells treated with newly tested agents was verified by the detection of LC3 puncta and conversion of LC3I to LC3II form, the phenomena related to autophagosome formation. The immunofluorescence output demonstrated the presence of LC3 puncta in GB cells treated with ARA12 and ARA13, while CQ addition resulted in a block of autophagy process confirmed by the abundance of cells strongly positive for LC3 puncta (Figure 7a). Autophagic flux in GB cells treated with tested compounds was monitored by LC3-II turnover using Western blot analysis in the presence and absence of the lysosomal degradation inhibitor, chloroquine, according to guidelines of data interpretation [27,28,29]. The levels of LC3-II (normalized to beta-actin) were higher in the samples treated with a combination of ARA12 or ARA13 with CQ than the amounts of LC3-II detected in the samples treated with single agents, providing evidence of autophagic flux stimulation by ARA12 and ARA13. A similar phenomenon, however less intense, was observed in cells treated with TMZ (Figure 7b).

Data obtained from Western blot regarding LC3II protein status were concurrent with output from immunofluorescent staining of said protein (Figure 7) and results generated by means of autophagy detection assay (Figure 6). The latter technique is independent of the detection of protein markers of autophagy and based on selective staining of pre-autophagosomes, autophagosomes, and autolysosomes with a cationic amphiphilic tracer dye. Altogether, autophagic flux in GB cells treated with TMZ, ARA12, and ARA13 was more prominent than in untreated cells. The effect was visualized independently with methods involving different means of detecting autophagy.

**Figure 8:**
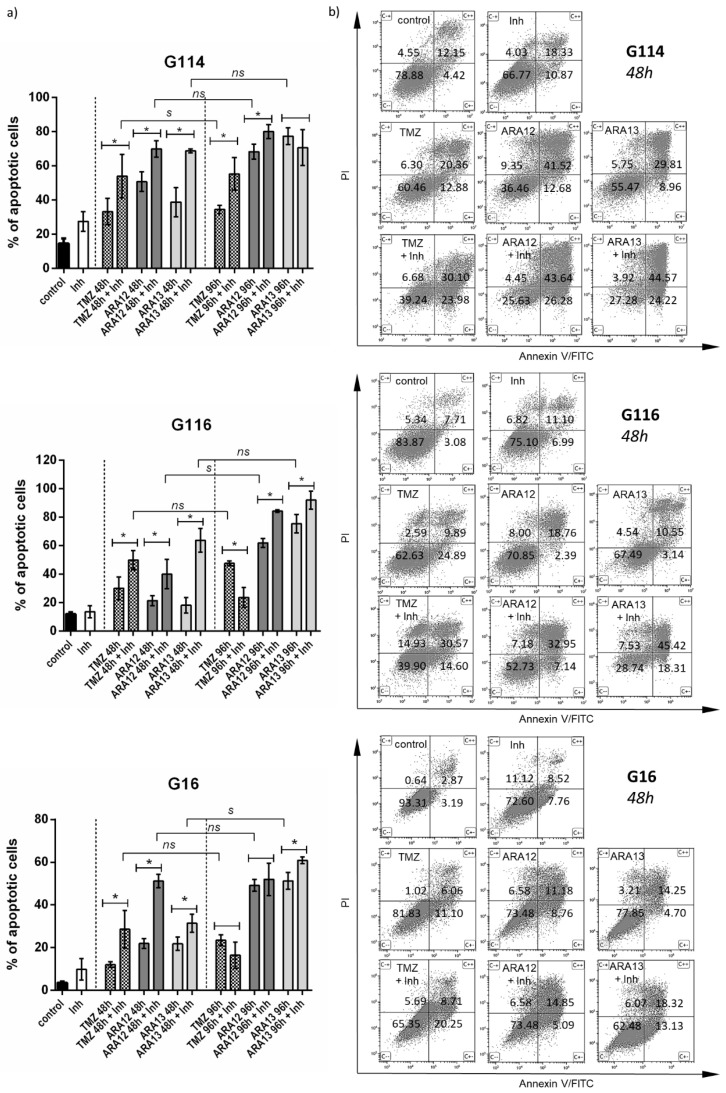
Inhibition of treatment-induced autophagy by chloroquine facilitate apoptosis initiated by TMZ and new aziridine–hydrazide hydrazone derivatives—ARA12 and ARA13. (**a**) Quantitative analysis of FACS data revealed a significant increase in apoptosis efficiency in samples treated with combination of a particular tested agent (ARA12, ARA13, or TMZ) with an autophagy inhibitor (Inh) after 48 h (*) when percentage of apoptotic cells was close to the values detected after a 96-h treatment with a single compound (s—differences significant, ns—differences non-significant); *p* < 0.05. (**b**) Results of FACS analysis show response of G114, G116, and G16 tumors to the tested agents (TMZ, ARA12, ARA13) alone or in combination with CQ (Inh) after 48-h treatment (mean percentage values of cells for distinguished populations were provided).

Subsequently, we verified if autophagy block influenced apoptosis initiated by aziridine–hydrazide hydrazone derivatives and TMZ in GB cells. The output of the apoptotic assay demonstrated that the addition of autophagy inhibitor to the tested compounds resulted in a significant increase in apoptosis efficiency after 48- and 96-h treatments (*p* < 0.05). However, the effect was stronger in the initial period. For 48-h incubation, the number of apoptotic cells was significantly higher for treatment comprising CQ addition in all tumors (*p* < 0.05), giving an increase in apoptotic cells count by 10–21% for TMZ with CQ (20.84%—G114, 10.39%—G116, 12.57%—G16), 15–31% for ARA12 with CQ (15.72%—G114, 18.94%—G116, 31.32%—G16) and 12–50% for ARA13 with CQ (30.02%—G114, 50.05%—G116, 12.50%—G16), however, differences in response between individual tumors were observed (table with *p* values for individual tumors were provided in Supplementary Material—Table S2). The percentage of apoptotic cells in samples exposed to 48-h combined treatment and in samples undergoing 96-h treatment with a single agent showed no statistically significant difference when compared, except for TMZ treatment in G114, ARA12 treatment in G116 and ARA13 treatment in G16. The apoptotic cells percentage values were 30–54% for 48 h TMZ with CQ and 23–47% for 96 h TMZ, 40–70% for 48h ARA12 with CQ, and 49–68% for 96h ARA 12, 30–68% for 48 h ARA13 with CQ and 51–75% for ARA 13. In the case of a 96-h incubation, the differences between tumors’ responses to a particular mode of treatment were more pronounced (Figure 8).

Analysis of the percentage of cells classified as early and late apoptotic after a particular mode of treatment with both new aziridine–hydrazide hydrazone derivatives demonstrated that the addition of CQ facilitates apoptosis causing more cells to enter the early phase of this process after 48 h of combined treatment (Figure 8). ARA12 treatment combined with CQ resulted in 2- to 3-fold increase in the early apoptotic population of G116 and G114 and even an 8.5-fold increase in G16. ARA13-CQ combined treatment yielded close to three-fold enrichment of early apoptotic fraction in the case of G16 and G114 tumors, while for G116, a four- to five-fold increase in both the early and late apoptotic populations was noted. The strength of the final effect resulted in a decrease in cell viability and was dependent on individual tumor responsiveness. A similar tendency was observed for TMZ treatment; however, the effect appeared to be weaker (Table 1).

**Figure 9:**
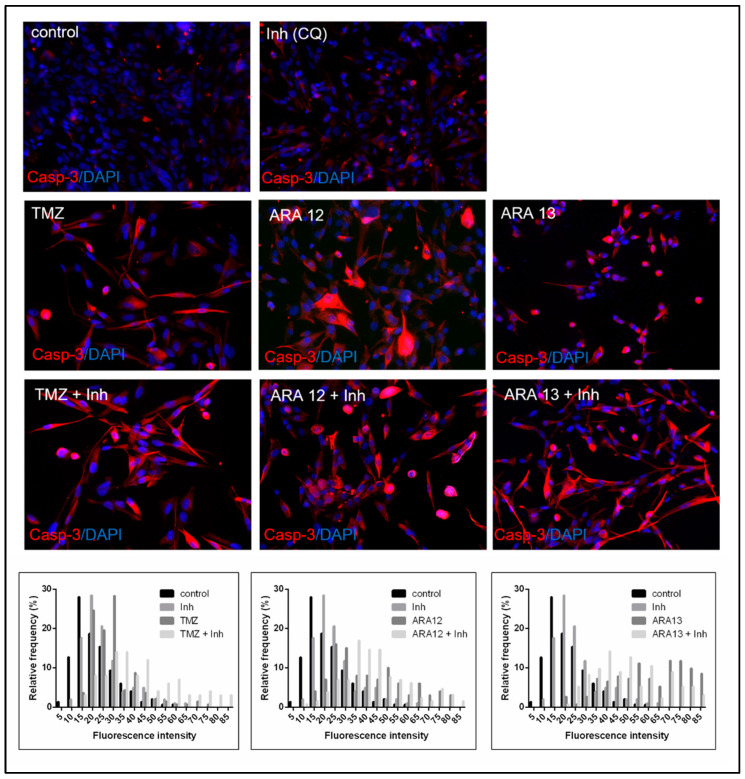
Caspase-3 activity status in GB cells after a 48-h treatment with TMZ, ARA12, ARA13, and combination of the tested agents with an autophagy inhibitor–chloroquine (Inh). Immunofluorescence results performed with antibody exclusively recognizing cleaved caspase-3 demonstrated that induction of apoptosis with ARA12 or ARA13 activated caspase-3 in GB cells, while addition of the autophagy inhibitor (CQ) enhanced apoptotic process, which was proven by the fact that the majority of cells appeared to be positive for cleaved caspase-3. A similar phenomenon was detected for cells treated with TMZ alone or in combination with chloroquine. The semiquantitative analysis of immunofluorescence data is presented as histograms visualizing changes in the distribution of cells presenting different levels of caspase-3 activity in response to treatment mode.

Since activation of caspases is one of the best-recognized biochemical hallmarks of both the early and late stages of apoptosis, the pro-apoptotic activity of the tested compounds was additionally verified by the detection of the cleaved form of caspase 3, being the key executioner protein. Immunofluorescence assay performed with the use of an antibody recognizing only the cleaved form of caspase-3 demonstrated that both ARA12 and ARA13 were able to initiate apoptosis in GB cells, thus leading to activation of caspase-3 within 48 h. Simultaneous treatment with a particular tested agent and CQ yielded results cohesive with FC experiments, showing enhancement of apoptosis, the phenomenon confirmed by the presence of GB cells expressing high levels of cleaved caspase-3 (Figure 9).

## 4. Discussion

Despite a spectrum of potential innovative methods of anti-neoplastic treatment announced in recent papers, glioblastoma remains practically incurable. Apart from possible modern options of anti-tumor treatments, like molecular targeted therapy or immunotherapy, chemotherapeutics presenting selective cytotoxic or genotoxic potential are still being searched for [33,34].

Among the abundance of examined compounds, the hydrazone- or aziridine derivatives appeared to have anti-neoplastic properties. Aziridine-containing compounds are known for their alkylating potential and ability to interstrand cross-linking formation in DNA—the genotoxic mechanisms underlying their biological activity against cancer cells. Hydrazone-based agents present a diverse spectrum of actions influencing cell cycle and cell death pathways (e.g., caspase activation); however, their cellular targets and final anti-neoplastic effect seem to depend on the chemical structure of particular derivatives. What is interesting, some of the bioactive NAH-derived scaffolds show broad synergy with conventional chemotherapeutic agents, including temozolomide [12,13,15,16,17,18,21]. Our study examined the newly synthesized class of compounds based on a combination of both aziridine- and hydrazide hydrazone derivatives in one molecule. Initial screening on tumor cells revealed the cytotoxic potential of ARA12 and ARA13—the two of several chemical variants that have been tested.

Further results clearly showed the pro-apoptotic potential of the two selected compounds, ARA12 and ARA13, against glioblastoma cells. The three GB-derived in vitro models presented higher sensitivity to the tested compounds than normal human astrocytes. However, some differences in the response of individual tumors were observed. Those discrepancies may have resulted from the molecular and phenotypic heterogeneity typical for GBs, but in the end, the final effectiveness of 96-h treatment for all tumors was higher than the status detected after TMZ application, irrespective of their inherent differences in drug sensitivity. Despite the fact that TMZ is still a gold standard in GB therapy, developing tumor resistance to this agent is a common reason for disease recurrence [9].

Although TMZ has the ability to inhibit the proliferation of GB cells and stimulate the process of their death, it has been recognized that TMZ treatment induces several mechanisms hindering apoptosis, including pro-survival autophagy, the phenomenon also confirmed by our current results [35,36].

Cytoprotective autophagy is an undesired side effect often induced by antineoplastic treatment; thus, it is considered a therapeutic target to overcome tumor resistance and execute cell death. However, autophagy in cancer is recognized as a double-edged sword, as some medications may exert a direct cytotoxic effect via an autophagic cell death pathway, despite the pro-survival face of this phenomenon [37,38,39].

Notwithstanding, recent in vitro and in vivo findings demonstrated that employing autophagy inhibitors in protocols of anti-cancer treatment increases the effectiveness of treatments. Chloroquine is one of the most common agents used to impair the autophagic process [40,41]. The usefulness of CQ as a blocker of autophagy induced by TMZ was also confirmed by our results.

The new aziridine–hydrazide hydrazone derivatives (ARA12 and ARA13) also appeared to be stimulants of autophagy in GB cells within the initial period of treatment (48 h); thus, we adopted a similar experimental approach to determine if an autophagy block can facilitate apoptosis process in GB cells. The output of autophagy and apoptosis assays demonstrated the inhibitory activity of CQ that resulted in increased efficiency of cell death induced by the tested agents.

Since the mechanism of the cytotoxic action of aziridine–hydrazide hydrazone derivatives remains unknown, we evaluated the status of caspase-3 activity as a key protein of the executioner phase of cell death to visualize the proapoptotic activity of the examined agents. One of the well-known compounds with NAH motif scaffolds had been recognized previously as an activator of pro-caspase 3 via the chelation of inhibitory zinc, which leads to its autocatalytic activation [21,42,43]. Our results of immunocytofluorescence assay, showing the presence of GB cells positive for cleaved caspase-3 after 48-h treatment with ARA12 or ARA13, were cohesive with quantitative FACS analysis based on annexin V/PI staining, as well as with results showing the occurrence of DNA fragmentation detected as subG0/G1 population and clearly demonstrate the ability of tested agents to stimulate apoptotic cell death.

Interestingly, in some research, an active form of caspase-3 is recognized as a protein that may play a role in a switch between autophagy and apoptosis, promoting the latter [43,44]. Thus, the abundance of cells positive for cleaved caspase-3 in GB treated with a combination of the tested agents and CQ may be considered as an endorsement of enhanced apoptosis, which was also confirmed by FACS analysis used to compare the progress of apoptosis in relation to treatment mode. Similarly to Lee et al., we did not observe increased activity of caspase-3 in cells treated with CQ alone [45]. Nevertheless, the activation of caspase-3 can not be treated as a sole indicator of autophagy inhibition, as the relationship between caspase-3 action and autophagy appears to be more complex and involves plenty of interlinked processes [46].

Our study is the first to show the anti-neoplastic activity of the newly synthesized aziridine–hydrazide hydrazone derivatives, thus certain issues remain to be elucidated at the level of the cellular model, like recognizing the molecular mechanism of the tested compounds’ action or the reason for different sensitivity presented by individual tumors. However, the output of our analyses clearly demonstrates the proapoptotic activity of the tested compounds against practically incurable brain tumors, glioblastoma, although ARA12 seems to be a more promising candidate for further investigation, considering its higher selectivity against neoplastic cells. Since the examined agents include two types of chemical moieties with proven bioactivity against cancer cells, the mechanisms underlying their cytotoxic potential can be more complex and involve different cellular pathways. Anti-tumor activity of the tested compounds may also be enhanced by the presence of fluorine atoms in their structure, especially since the initially screened variants, devoid of these fluorinated moieties, did not show so strong cytotoxic activity against GB cells (and fluoro-pharmaceuticals are known for their bioactivity including anti-neoplastic properties [47]. Despite the fact that aziridine- or hydrazone-based chemicals are extensively investigated as candidate medicines in a broad spectrum of diseases, and some of them have been introduced in the market, further research is needed to characterize the pharmacological properties of our newly synthesized molecules since their complex chemical nature may not only enhance the anti-tumor potential but also influence their safety profile.

One of the causes of glioblastoma drug resistance appears to be the blood–brain barrier (BBB) insulating neoplastic cells from applied chemotherapeutics, especially as combined therapy may include anti-angiogenic drugs which further decrease the barrier’s permeability [48,49].

An algorithm designed by Gupta et al. for predicting the probability of the drug penetrating the blood–brain barrier based on a dataset of 940 drug molecules was employed for preliminary assessment of the ability of new aziridine–hydrazide hydrazone derivatives to cross BBB.

Although the results of the BBB Score obtained for ARA12 (3.66) and ARA13 (3.40) did not reach the top values presented by the majority of tested CNS drugs (BBB score 4–5), they do not exclude them from further investigation, since the authors claim that about 20% of drugs with BBB Scores between 3 and 4 are routinely used as CNS therapeutics [30]. The fact that preliminary assessed BBB Scores for tested compounds are shared with other drugs already used in central nervous system therapy justifies conducting further, more advanced research aimed at evaluating compounds’ usefulness as potential candidates for CNS therapeutics. Therefore, at the stage of ex vivo studies, we plan to verify their permeation via BBB with the use of a transwell system model of BBB supported with complex in silico analyses [50].

The BBB remains an impenetrable obstacle for many new promising antineoplastic agents and limits the effectiveness of plenty of drugs delivered through the systemic route. The main alternatives to systemic route delivery comprise delivering the drug directly to the brain tissue through surgical intervention by cerebrospinal fluid, convection-enhanced delivery, or implants containing therapeutics [51,52,53]. The other examined approach is focused on enhancing the bioavailability of therapeutics in the brain tissue by facilitating their way through the blood–brain barrier. Those include increasing compounds liposolubility by liposome encapsulation and modulating the blood–brain barrier itself by inhibiting efflux pumps, increasing blood–brain permeability with bradykinin receptors adenosine receptors activation, treating tight junctions physically, for example, with ultrasounds or osmotic agents [53,54,55]. Currently, engineered nanomaterials can encapsulate therapeutic compounds in a shell with programmable size, charge, and surface biochemistry, which allows for exploiting blood–brain barrier transportation pathways [56]. A variety of nanocarriers, such as polymeric nanoparticles, liposomes, lipid nanoparticles, dendrimers, or inorganic nanoparticles, have been formulated to overcome BBB and subjected to clinical trials [57]. Also, molecular carriers like cryptands, calixarenes, cyclophanes, spherands, cyclodextrins, and crown ethers, which can form intermolecular interactions with small molecules based on the “host–guest” relation, have been investigated as potential devices allowing for effective drug delivery [58]. Those types of supramolecular structures based on cyclodextrin cages, with the proven ability to cross the BBB, could be utilized as potential carriers of new aziridine–hydrazide hydrazone derivatives (ARA12 and ARA13) [59]. Presented compounds have the right substituents and are the appropriate size to form hydrogen bonds and adapt to such molecular carriers. An alternative option is linking the tested compounds to BBB-penetrating peptide shuttles so as to create peptide–drug conjugates able to get through the BBB by using the peptides with BBB-penetrating properties as vectors to deliver small therapeutic molecules to brain parenchyma [60]. This approach will require the use of a suitable ester linker but is still possible.

Additionally, the carriers can be modified to selectively target tumoral cells in order to minimize the undesired side effects of injuring the non-neoplastic microenvironment. Il13Rα2 receptor specific for astrocytoma cells had been previously investigated as a route to specific delivery of drugs to GB cells based on different types of nanocarriers conjugated with IL13 homing-peptide being a ligand for Il13Rα2 [23,61,62,63,64,65,66].

Although our results demonstrated the different sensitivity of GB and normal cells (NHA) to tested agents, the compounds are not neutral for non-neoplastic cells. Thus, we will further examine the optimal effective treatment dosage in vitro using the co-culture of GB with NHA cells. To minimize the cytotoxic effect on normal cells, we will test an alternative method of targeted drug delivery with the use of Il13-peptide-conjugated innovative carriers (based on the cryptand-like supramolecular complex type of “host-guest”) for delivery of tested compounds to GB cells expressing Interleukin- 13 rα2.

Moreover, our findings proved the importance of pro-survival autophagy as an undesired effect of treatment that impairs the execution of cell death. This phenomenon seems to be underrated, though it is already at the stage of new candidate drug testing, which may lead to premature abandonment of research. Thus, the standard search for new chemotherapeutics could be more effective if accompanied by an analysis of the mechanisms of tumor resistance to tested agents. Such an experimental approach could initially verify if tested compounds are able to stimulate undesired processes responsible for therapy escape and treatment failure.

Extrapolating our findings from bench to clinic, we postulate that including an autophagy inhibitor in the treatment protocol would constitute great assistance in designing a more effective therapy, not only by facilitating the death of neoplastic cells but also by minimizing the risk of tumor therapy escape.

Additionally, implementing treatment with an optimal combination of an apoptosis inducer and an autophagy inhibitor would create a chance to decrease the concentration of a chemotherapeutic or shorten treatment periods, which seems to be important, especially in the case of highly cytotoxic drugs presenting limited selectivity to tumor cells.

## Figures and Tables

**Table 1:** Influence of autophagy inhibitor on progress of apoptotic process and viability of GB cells after 48-h treatment with TMZ, ARA12, and ARA13.

Treatment Mode	G16			G116			G114		
	Early Apopt.	Late Apopt.	Viable Cells (%)	Early Apopt.	Late Apopt.	Viable Cells (%)	Early Apopt	Late Apopt.	Viable Cells (%)
	(combined/single)			(combined/single)			(combined/single)		
untreated	-	-	93.31 ± 2.69	-	-	83.87 ± 4.99	-	-	78.88 ± 4.81
TMZ	↑ (1.83)	↑ (1.62)	82.62 ± 6.32	(0.58)	↑↑ (3.09)	62.63 ± 8.85	↑ (1.68)	↑ (1.58)	60.46 ± 3.59
TMZ + Inh			65.35 ± 8.65 *			39.90 ±13.41 *			39.24 ± 10.54 *
ARA12	↑↑↑ (8.50)	(0.53)	73.48 ± 2.55	↑↑ (2.98)	↑ (1.75)	70.85 ± 3.88	↑↑ (2.07)	(1.05)	36.46 ± 6.44
ARA12 + Inh			43.31 ± 2.90 *			52.73 ± 7.18 *			25.63 ± 6.10 *
ARA13	↑↑ (2.79)	(1.26)	77.85 ± 6.28	↑↑↑ (5.83)	↑↑↑ (4.30)	67.49 ± 5.01	↑↑ (2.70)	↑ (1.50)	55.47 ± 9.96
ARA13 + Inh			62.48 ± 5.69 *			28.74 ± 7.53 *			27.28 ± 1.49 *

Evaluation of CQ influence on apoptotic population enrichment (↑—weak effect, 1.5–2-fold increase; ↑↑—moderate effect, 2–4-fold increase; ↑↑↑—strong effect, >4-fold increase) and GB cells viability compared to cells treated with single agent (*); *p* < 0.05.

## Data Availability

All data generated or analyzed during this study are included in this article.

## Supplementary Materials

The following supporting information can be downloaded at: https://www.mdpi.com/article/10.3390/cells12141906/s1, Table S1: Influence of tested compounds on progress of apoptotic process and viability of GB cells after 24-h treatment; Table S2: Comparison of percentage of apoptotic cells after combined and single treatment mode (results of statistical analysis for individual tumors); Figure S1: Results of Western blot analysis for LC3 protein (uncropped membranes); Figure S2: Results of apoptosis assay (FACS analysis) for NHA cells treated with ARA12.
