# Supplementary material for: Autophagy Inhibition with Chloroquine Increased Pro-Apoptotic Potential of New Aziridine-Hydrazide Hydrazone Derivatives against Glioblastoma Cells
Source: Cells. 2023 Jul 21;12(14):1906. doi: 10.3390/cells12141906 (PMC10378024; doi:10.3390/cells12141906)

Supplementary materials

Table S1

Influence of tested compounds on progress of apoptotic process and viability of GB cells after 24-hour treatment

Treatment mode	G16			G114			G116		
	Early apopt.	*Late apopt.*	*Viable cells [%]*	*Early apopt.*	*Late apopt.*	*Viable cells [%]*	*Early apopt.*	*Late apopt.*	*Viable cells [%]*
**Control**	1.86 ±0.13	7.69±1.05	88.2±4.69	3.67±0.71	8.45±0.43	85.59±4.78	0.86±0.10	7.47±0.53	87.06±3.36
**ARA12**	10.59±0.74	15.34±1.4	71.76±1.88	1.23±0.14	6.38±0.48	89.46±0.54	4.38±1.42	5.95±1.15	85.59±1.49
**ARA13**	15.39±2.44	13.7±1.27	69.17±3.85	3.84±0.35	5.53±0.56	88.37±1.2	2.77±0.81	5.16±0.4	89.14±0.91

Table S2

Comparison of percentage of apoptotic cells after combined and single treatment mode
(results of statistical analysis for individual tumors; *p* values).

Adjusted p values		**TMZ vs TMZ+Inh**	**ARA12 vs ARA12+Inh**	**ARA13 vs ARA13+Inh**
**G114**	48h	0.038	0.0009	0.0004
	96h	0.031	0.0134	ns (*p*>0.05)
**G116**	48h	0.0019	0.0002	<0.0001
	96h	0.0004	<0.0001	0.0093
**G16**	48h	0.0041	<0.0001	0.0066
	96h	ns (*p*>0.05)	ns (*p*>0.05)	0.0066

Figure S1

Results of Western blot analysis for LC3 protein (uncropped membranes).

G 116

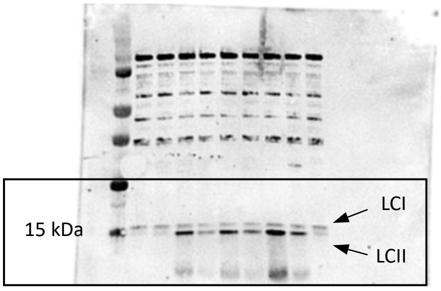

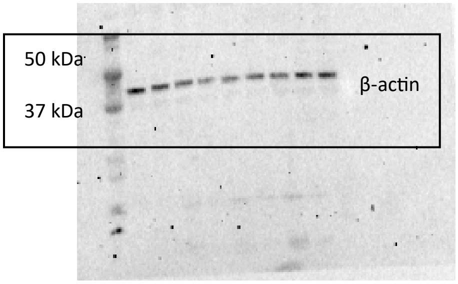

G16

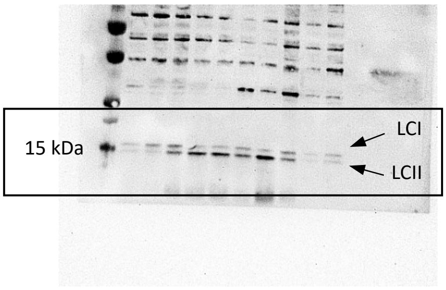

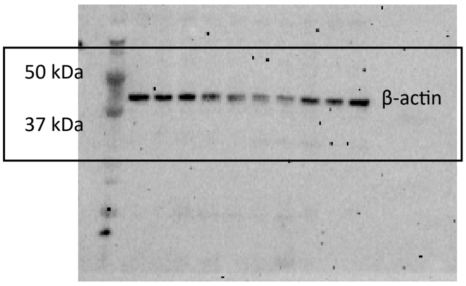

Figure S2

Results of apoptosis assay performed via FACS analysis for NHA treated with ARA12 [150µg/mL].

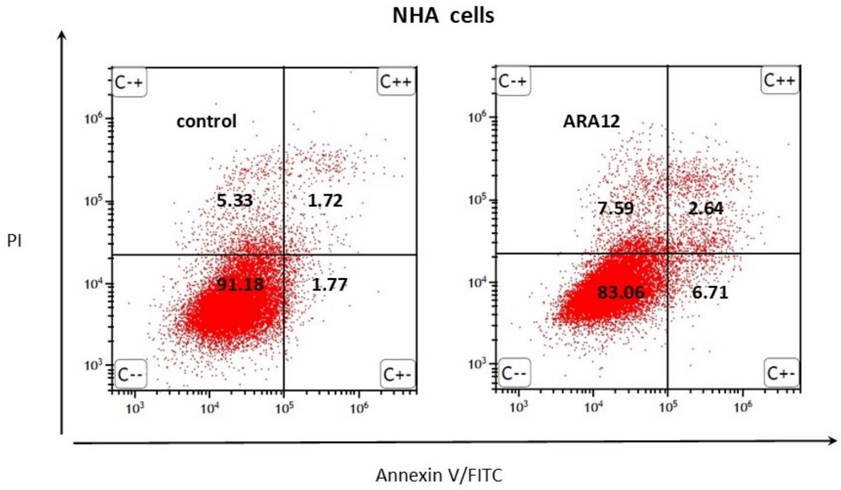

Supplement: Supplementary file 1 [file cells-12-01906-s001.zip › cells-2432354-supplementary.pdf]
